# Supplementary material for: Modeling glioblastoma heterogeneity as a dynamic network of cell states
Source: Mol Syst Biol. 2021 Sep 16;17(9):e10105. doi: 10.15252/msb.202010105 (PMC8444284; doi:10.15252/msb.202010105)
Supplement: Supplementary file 6 — Source Data for Figure 5 [file MSB-17-e10105-s004.zip › Figure5A_sourcedata/GSEA_3071/nosigdb_stateI.GseaPreranked.1622795145664/RICHARDS_NOWAKOWSKI_SCIENCE_2017_MGE_DIV_UPREG.html]

Details for gene set RICHARDS\_NOWAKOWSKI\_SCIENCE\_2017\_MGE\_DIV\_UPREG[GSEA]

|  || Dataset | state13071 |
| Phenotype | NoPhenotypeAvailable |
| Upregulated in class | na\_neg |
| GeneSet | RICHARDS\_NOWAKOWSKI\_SCIENCE\_2017\_MGE\_DIV\_UPREG |
| Enrichment Score (ES) | -0.53991807 |
| Normalized Enrichment Score (NES) | -3.4087307 |
| Nominal p-value | 0.0 |
| FDR q-value | 0.0 |
| FWER p-Value | 0.0 |
Table: GSEA Results Summary

  

Fig 1: Enrichment plot: RICHARDS\_NOWAKOWSKI\_SCIENCE\_2017\_MGE\_DIV\_UPREG      
 Profile of the Running ES Score & Positions of GeneSet Members on the Rank Ordered List

  

| PROBE | GENE SYMBOL | GENE\_TITLE | RANK IN GENE LIST | RANK METRIC SCORE | RUNNING ES | CORE ENRICHMENT || 1 | BICC1 |  |  | 38 | 1.463 | -0.0063 | No |
| 2 | OMD |  |  | 75 | 1.196 | -0.0131 | No |
| 3 | SLC20A1 |  |  | 116 | 1.035 | -0.0218 | No |
| 4 | FAM114A1 |  |  | 174 | 0.864 | -0.0367 | No |
| 5 | STK17B |  |  | 274 | 0.707 | -0.0658 | No |
| 6 | PHGDH |  |  | 279 | 0.699 | -0.0643 | No |
| 7 | HSPA5 |  |  | 337 | 0.638 | -0.0801 | No |
| 8 | MYO1B |  |  | 441 | 0.556 | -0.1111 | No |
| 9 | TALDO1 |  |  | 455 | 0.545 | -0.1131 | No |
| 10 | TMEM98 |  |  | 510 | 0.502 | -0.1285 | No |
| 11 | SEC11A |  |  | 518 | 0.496 | -0.1287 | No |
| 12 | IQGAP1 |  |  | 521 | 0.494 | -0.1274 | No |
| 13 | PDLIM3 |  |  | 547 | 0.481 | -0.1335 | No |
| 14 | IGF2BP1 |  |  | 591 | 0.457 | -0.1455 | No |
| 15 | HDLBP |  |  | 696 | 0.413 | -0.1774 | No |
| 16 | PARP4 |  |  | 705 | 0.410 | -0.1784 | No |
| 17 | PKD2 |  |  | 754 | 0.396 | -0.1923 | No |
| 18 | SHMT2 |  |  | 777 | 0.390 | -0.1978 | No |
| 19 | CLIC4 |  |  | 800 | 0.382 | -0.2033 | No |
| 20 | ARHGEF40 |  |  | 899 | 0.351 | -0.2336 | No |
| 21 | UPF2 |  |  | 1016 | 0.325 | -0.2697 | No |
| 22 | SERINC5 |  |  | 1066 | 0.313 | -0.2843 | No |
| 23 | LRIG2 |  |  | 1168 | 0.295 | -0.3157 | No |
| 24 | CCDC34 |  |  | 1376 | 0.255 | -0.3816 | No |
| 25 | MSN |  |  | 1379 | 0.255 | -0.3812 | No |
| 26 | SLC7A1 |  |  | 1389 | 0.254 | -0.3830 | No |
| 27 | TMBIM6 |  |  | 1391 | 0.253 | -0.3823 | No |
| 28 | RAP1B |  |  | 1400 | 0.252 | -0.3839 | No |
| 29 | NDUFA6 |  |  | 1420 | -0.250 | -0.3890 | No |
| 30 | MSH2 |  |  | 1438 | -0.252 | -0.3935 | No |
| 31 | CEP152 |  |  | 1446 | -0.253 | -0.3947 | No |
| 32 | GAPDH |  |  | 1459 | -0.253 | -0.3976 | No |
| 33 | UQCRQ |  |  | 1464 | -0.254 | -0.3978 | No |
| 34 | SRSF10 |  |  | 1489 | -0.256 | -0.4045 | No |
| 35 | HNRNPH1 |  |  | 1500 | -0.257 | -0.4067 | No |
| 36 | ZGRF1 |  |  | 1505 | -0.258 | -0.4070 | No |
| 37 | CTPS1 |  |  | 1540 | -0.261 | -0.4169 | No |
| 38 | RBBP4 |  |  | 1549 | -0.262 | -0.4184 | No |
| 39 | RPLP0 |  |  | 1551 | -0.262 | -0.4177 | No |
| 40 | POLE2 |  |  | 1560 | -0.263 | -0.4192 | No |
| 41 | VSIG10 |  |  | 1563 | -0.263 | -0.4188 | No |
| 42 | ARL6IP6 |  |  | 1577 | -0.265 | -0.4219 | No |
| 43 | SUPT16H |  |  | 1588 | -0.266 | -0.4240 | No |
| 44 | ERI2 |  |  | 1593 | -0.267 | -0.4242 | No |
| 45 | C4orf46 |  |  | 1596 | -0.267 | -0.4238 | No |
| 46 | TTK |  |  | 1620 | -0.270 | -0.4301 | No |
| 47 | MBD4 |  |  | 1621 | -0.270 | -0.4290 | No |
| 48 | MPHOSPH9 |  |  | 1623 | -0.270 | -0.4282 | No |
| 49 | STAG1 |  |  | 1624 | -0.270 | -0.4271 | No |
| 50 | IPO9 |  |  | 1634 | -0.272 | -0.4290 | No |
| 51 | HNRNPF |  |  | 1636 | -0.272 | -0.4282 | No |
| 52 | SAE1 |  |  | 1638 | -0.273 | -0.4274 | No |
| 53 | SMC3 |  |  | 1650 | -0.274 | -0.4298 | No |
| 54 | PPAT |  |  | 1655 | -0.275 | -0.4300 | No |
| 55 | RNASEH2C |  |  | 1656 | -0.275 | -0.4289 | No |
| 56 | SNX5 |  |  | 1663 | -0.275 | -0.4297 | No |
| 57 | BUB3 |  |  | 1687 | -0.278 | -0.4360 | No |
| 58 | RAD54L |  |  | 1702 | -0.280 | -0.4394 | No |
| 59 | YBX1 |  |  | 1725 | -0.283 | -0.4453 | No |
| 60 | KIF2C |  |  | 1735 | -0.284 | -0.4471 | No |
| 61 | SACS |  |  | 1738 | -0.285 | -0.4466 | No |
| 62 | NOSIP |  |  | 1744 | -0.286 | -0.4470 | No |
| 63 | PSMC3 |  |  | 1752 | -0.287 | -0.4481 | No |
| 64 | CCNB2 |  |  | 1757 | -0.288 | -0.4482 | No |
| 65 | ZNF273 |  |  | 1760 | -0.288 | -0.4477 | No |
| 66 | CALM2 |  |  | 1763 | -0.289 | -0.4472 | No |
| 67 | MIS18BP1 |  |  | 1764 | -0.289 | -0.4460 | No |
| 68 | CENPJ |  |  | 1768 | -0.290 | -0.4458 | No |
| 69 | EXOSC9 |  |  | 1777 | -0.291 | -0.4472 | No |
| 70 | HNRNPA2B1 |  |  | 1819 | -0.296 | -0.4592 | No |
| 71 | TPI1 |  |  | 1828 | -0.297 | -0.4606 | No |
| 72 | RHOA |  |  | 1843 | -0.298 | -0.4639 | No |
| 73 | CASP8AP2 |  |  | 1857 | -0.300 | -0.4669 | No |
| 74 | CENPQ |  |  | 1860 | -0.300 | -0.4663 | No |
| 75 | TTF2 |  |  | 1873 | -0.301 | -0.4690 | No |
| 76 | RNF138 |  |  | 1894 | -0.304 | -0.4742 | No |
| 77 | RAN |  |  | 1907 | -0.306 | -0.4768 | No |
| 78 | RPL7L1 |  |  | 1908 | -0.306 | -0.4756 | No |
| 79 | IQCB1 |  |  | 1929 | -0.309 | -0.4808 | No |
| 80 | COPS6 |  |  | 1949 | -0.312 | -0.4857 | No |
| 81 | EXOG |  |  | 1952 | -0.313 | -0.4850 | No |
| 82 | EIF1AY |  |  | 2003 | -0.317 | -0.4999 | No |
| 83 | SUZ12 |  |  | 2011 | -0.318 | -0.5009 | No |
| 84 | PARPBP |  |  | 2026 | -0.322 | -0.5041 | No |
| 85 | ANAPC11 |  |  | 2037 | -0.323 | -0.5060 | No |
| 86 | DDX39A |  |  | 2043 | -0.324 | -0.5063 | No |
| 87 | ACYP1 |  |  | 2052 | -0.326 | -0.5076 | No |
| 88 | DNA2 |  |  | 2057 | -0.327 | -0.5075 | No |
| 89 | SFMBT1 |  |  | 2061 | -0.327 | -0.5072 | No |
| 90 | SNRPB |  |  | 2064 | -0.328 | -0.5065 | No |
| 91 | MZT2B |  |  | 2075 | -0.330 | -0.5084 | No |
| 92 | CCDC15 |  |  | 2080 | -0.331 | -0.5083 | No |
| 93 | E2F7 |  |  | 2081 | -0.331 | -0.5070 | No |
| 94 | ZNF93 |  |  | 2095 | -0.333 | -0.5098 | No |
| 95 | CHEK1 |  |  | 2108 | -0.336 | -0.5123 | No |
| 96 | ZNF367 |  |  | 2131 | -0.339 | -0.5180 | No |
| 97 | BRIX1 |  |  | 2135 | -0.340 | -0.5176 | No |
| 98 | CORO1C |  |  | 2136 | -0.340 | -0.5162 | No |
| 99 | HNRNPDL |  |  | 2142 | -0.342 | -0.5165 | No |
| 100 | RPL8 |  |  | 2154 | -0.344 | -0.5186 | No |
| 101 | PLK4 |  |  | 2167 | -0.346 | -0.5211 | No |
| 102 | KIAA1958 |  |  | 2170 | -0.347 | -0.5203 | No |
| 103 | ADK |  |  | 2186 | -0.349 | -0.5238 | No |
| 104 | YEATS4 |  |  | 2187 | -0.349 | -0.5223 | No |
| 105 | DLEU2 |  |  | 2196 | -0.351 | -0.5235 | No |
| 106 | GLCCI1 |  |  | 2212 | -0.356 | -0.5269 | No |
| 107 | ILF3 |  |  | 2222 | -0.358 | -0.5283 | No |
| 108 | TOMM5 |  |  | 2224 | -0.358 | -0.5272 | No |
| 109 | SMC1A |  |  | 2240 | -0.361 | -0.5306 | No |
| 110 | SNRPE |  |  | 2247 | -0.362 | -0.5310 | No |
| 111 | POLE |  |  | 2252 | -0.364 | -0.5309 | No |
| 112 | POLQ |  |  | 2253 | -0.364 | -0.5294 | No |
| 113 | NUCKS1 |  |  | 2278 | -0.368 | -0.5356 | No |
| 114 | MTHFD1 |  |  | 2282 | -0.369 | -0.5351 | No |
| 115 | DEPDC1B |  |  | 2288 | -0.370 | -0.5352 | No |
| 116 | TUBB4B |  |  | 2291 | -0.370 | -0.5344 | No |
| 117 | GLO1 |  |  | 2292 | -0.371 | -0.5329 | No |
| 118 | TIMM10 |  |  | 2296 | -0.372 | -0.5323 | No |
| 119 | MSI2 |  |  | 2314 | -0.375 | -0.5363 | No |
| 120 | SRP9 |  |  | 2319 | -0.375 | -0.5360 | No |
| 121 | CKS1B |  |  | 2332 | -0.380 | -0.5384 | Yes |
| 122 | NOP56 |  |  | 2333 | -0.380 | -0.5368 | Yes |
| 123 | BUB1B |  |  | 2335 | -0.381 | -0.5356 | Yes |
| 124 | MCM2 |  |  | 2349 | -0.383 | -0.5382 | Yes |
| 125 | NUP50 |  |  | 2354 | -0.385 | -0.5380 | Yes |
| 126 | CKAP2 |  |  | 2357 | -0.385 | -0.5370 | Yes |
| 127 | ZWILCH |  |  | 2358 | -0.385 | -0.5355 | Yes |
| 128 | MSH6 |  |  | 2362 | -0.386 | -0.5349 | Yes |
| 129 | PSIP1 |  |  | 2364 | -0.386 | -0.5336 | Yes |
| 130 | CCNF |  |  | 2370 | -0.387 | -0.5337 | Yes |
| 131 | GLTP |  |  | 2371 | -0.388 | -0.5321 | Yes |
| 132 | STIL |  |  | 2374 | -0.388 | -0.5312 | Yes |
| 133 | POLA1 |  |  | 2376 | -0.388 | -0.5299 | Yes |
| 134 | H3F3A |  |  | 2377 | -0.388 | -0.5283 | Yes |
| 135 | RBMX |  |  | 2378 | -0.388 | -0.5267 | Yes |
| 136 | IPO5 |  |  | 2381 | -0.390 | -0.5258 | Yes |
| 137 | DDIAS |  |  | 2382 | -0.390 | -0.5242 | Yes |
| 138 | ABAT |  |  | 2383 | -0.390 | -0.5226 | Yes |
| 139 | MEA1 |  |  | 2386 | -0.390 | -0.5217 | Yes |
| 140 | HAUS6 |  |  | 2388 | -0.390 | -0.5204 | Yes |
| 141 | CSNK2B |  |  | 2391 | -0.390 | -0.5195 | Yes |
| 142 | RDX |  |  | 2399 | -0.392 | -0.5201 | Yes |
| 143 | CENPW |  |  | 2402 | -0.393 | -0.5192 | Yes |
| 144 | UHRF1 |  |  | 2404 | -0.393 | -0.5179 | Yes |
| 145 | PRIM2 |  |  | 2409 | -0.394 | -0.5176 | Yes |
| 146 | HAUS1 |  |  | 2423 | -0.397 | -0.5202 | Yes |
| 147 | WEE1 |  |  | 2426 | -0.397 | -0.5192 | Yes |
| 148 | H2AFV |  |  | 2431 | -0.398 | -0.5189 | Yes |
| 149 | HNRNPU |  |  | 2434 | -0.398 | -0.5179 | Yes |
| 150 | KCTD9 |  |  | 2438 | -0.399 | -0.5173 | Yes |
| 151 | ZNF738 |  |  | 2441 | -0.400 | -0.5163 | Yes |
| 152 | HNRNPA1 |  |  | 2455 | -0.402 | -0.5188 | Yes |
| 153 | UBE2N |  |  | 2458 | -0.403 | -0.5179 | Yes |
| 154 | SNRPG |  |  | 2459 | -0.404 | -0.5162 | Yes |
| 155 | CENPO |  |  | 2460 | -0.404 | -0.5146 | Yes |
| 156 | CKS2 |  |  | 2466 | -0.404 | -0.5145 | Yes |
| 157 | BLM |  |  | 2469 | -0.404 | -0.5135 | Yes |
| 158 | KIF11 |  |  | 2488 | -0.410 | -0.5177 | Yes |
| 159 | HAUS8 |  |  | 2494 | -0.412 | -0.5176 | Yes |
| 160 | CENPL |  |  | 2497 | -0.413 | -0.5166 | Yes |
| 161 | ZNF620 |  |  | 2503 | -0.416 | -0.5165 | Yes |
| 162 | CSE1L |  |  | 2512 | -0.417 | -0.5174 | Yes |
| 163 | SPAG5 |  |  | 2519 | -0.418 | -0.5176 | Yes |
| 164 | RACGAP1 |  |  | 2522 | -0.419 | -0.5166 | Yes |
| 165 | NFATC2IP |  |  | 2531 | -0.423 | -0.5174 | Yes |
| 166 | BAZ1B |  |  | 2538 | -0.425 | -0.5177 | Yes |
| 167 | PARP2 |  |  | 2542 | -0.425 | -0.5169 | Yes |
| 168 | FIGN |  |  | 2543 | -0.426 | -0.5152 | Yes |
| 169 | PAICS |  |  | 2545 | -0.426 | -0.5138 | Yes |
| 170 | NAP1L4 |  |  | 2552 | -0.427 | -0.5140 | Yes |
| 171 | TEX30 |  |  | 2556 | -0.428 | -0.5132 | Yes |
| 172 | WSB2 |  |  | 2560 | -0.429 | -0.5124 | Yes |
| 173 | NCAPD2 |  |  | 2563 | -0.431 | -0.5113 | Yes |
| 174 | CDK5RAP2 |  |  | 2564 | -0.431 | -0.5095 | Yes |
| 175 | H2AFZ |  |  | 2572 | -0.433 | -0.5100 | Yes |
| 176 | LRR1 |  |  | 2579 | -0.434 | -0.5102 | Yes |
| 177 | SSRP1 |  |  | 2580 | -0.434 | -0.5084 | Yes |
| 178 | TMEM106C |  |  | 2592 | -0.437 | -0.5102 | Yes |
| 179 | ANP32B |  |  | 2593 | -0.438 | -0.5084 | Yes |
| 180 | LDHB |  |  | 2597 | -0.438 | -0.5076 | Yes |
| 181 | PCDH17 |  |  | 2600 | -0.439 | -0.5065 | Yes |
| 182 | CIT |  |  | 2604 | -0.440 | -0.5057 | Yes |
| 183 | PHF10 |  |  | 2609 | -0.442 | -0.5052 | Yes |
| 184 | UBR7 |  |  | 2611 | -0.442 | -0.5037 | Yes |
| 185 | KNTC1 |  |  | 2613 | -0.443 | -0.5022 | Yes |
| 186 | RMI1 |  |  | 2616 | -0.444 | -0.5010 | Yes |
| 187 | SRSF7 |  |  | 2619 | -0.445 | -0.4999 | Yes |
| 188 | NCAPG2 |  |  | 2621 | -0.445 | -0.4984 | Yes |
| 189 | SRSF3 |  |  | 2631 | -0.447 | -0.4995 | Yes |
| 190 | HIRIP3 |  |  | 2635 | -0.449 | -0.4986 | Yes |
| 191 | LINC01224 |  |  | 2637 | -0.449 | -0.4971 | Yes |
| 192 | DUT |  |  | 2640 | -0.450 | -0.4959 | Yes |
| 193 | MASTL |  |  | 2646 | -0.452 | -0.4957 | Yes |
| 194 | SET |  |  | 2652 | -0.455 | -0.4955 | Yes |
| 195 | EXOSC8 |  |  | 2659 | -0.456 | -0.4956 | Yes |
| 196 | CCDC152 |  |  | 2664 | -0.460 | -0.4950 | Yes |
| 197 | RFWD3 |  |  | 2668 | -0.462 | -0.4941 | Yes |
| 198 | SKA2 |  |  | 2672 | -0.464 | -0.4931 | Yes |
| 199 | MND1 |  |  | 2682 | -0.468 | -0.4941 | Yes |
| 200 | KIF22 |  |  | 2683 | -0.468 | -0.4922 | Yes |
| 201 | CMC2 |  |  | 2694 | -0.470 | -0.4936 | Yes |
| 202 | CDCA8 |  |  | 2695 | -0.470 | -0.4916 | Yes |
| 203 | MMS22L |  |  | 2698 | -0.471 | -0.4904 | Yes |
| 204 | MCM6 |  |  | 2699 | -0.471 | -0.4885 | Yes |
| 205 | ANLN |  |  | 2702 | -0.472 | -0.4872 | Yes |
| 206 | HAT1 |  |  | 2703 | -0.472 | -0.4853 | Yes |
| 207 | PRC1 |  |  | 2705 | -0.472 | -0.4837 | Yes |
| 208 | FANCD2 |  |  | 2707 | -0.474 | -0.4821 | Yes |
| 209 | CENPN |  |  | 2708 | -0.474 | -0.4801 | Yes |
| 210 | MELK |  |  | 2709 | -0.474 | -0.4782 | Yes |
| 211 | SLC43A3 |  |  | 2713 | -0.475 | -0.4772 | Yes |
| 212 | TUBG1 |  |  | 2715 | -0.475 | -0.4756 | Yes |
| 213 | NDC80 |  |  | 2723 | -0.478 | -0.4759 | Yes |
| 214 | CYCS |  |  | 2727 | -0.479 | -0.4750 | Yes |
| 215 | EXO1 |  |  | 2728 | -0.479 | -0.4730 | Yes |
| 216 | RPA3 |  |  | 2730 | -0.480 | -0.4714 | Yes |
| 217 | PDZRN3 |  |  | 2731 | -0.480 | -0.4694 | Yes |
| 218 | SHCBP1 |  |  | 2733 | -0.480 | -0.4678 | Yes |
| 219 | BRIP1 |  |  | 2739 | -0.482 | -0.4675 | Yes |
| 220 | CHCHD3 |  |  | 2749 | -0.485 | -0.4684 | Yes |
| 221 | TIMELESS |  |  | 2752 | -0.487 | -0.4670 | Yes |
| 222 | CENPF |  |  | 2761 | -0.490 | -0.4676 | Yes |
| 223 | SLC25A5 |  |  | 2765 | -0.493 | -0.4666 | Yes |
| 224 | BRCA2 |  |  | 2772 | -0.495 | -0.4665 | Yes |
| 225 | NUF2 |  |  | 2781 | -0.497 | -0.4671 | Yes |
| 226 | SRSF2 |  |  | 2782 | -0.497 | -0.4651 | Yes |
| 227 | TUBA1B |  |  | 2785 | -0.498 | -0.4637 | Yes |
| 228 | SKA1 |  |  | 2787 | -0.498 | -0.4620 | Yes |
| 229 | SLBP |  |  | 2792 | -0.499 | -0.4612 | Yes |
| 230 | MGME1 |  |  | 2797 | -0.500 | -0.4605 | Yes |
| 231 | WDR76 |  |  | 2800 | -0.501 | -0.4591 | Yes |
| 232 | RAD18 |  |  | 2802 | -0.501 | -0.4574 | Yes |
| 233 | HMGB1 |  |  | 2806 | -0.503 | -0.4563 | Yes |
| 234 | RBL1 |  |  | 2808 | -0.503 | -0.4546 | Yes |
| 235 | COX8A |  |  | 2819 | -0.509 | -0.4557 | Yes |
| 236 | LSM5 |  |  | 2820 | -0.509 | -0.4537 | Yes |
| 237 | SIVA1 |  |  | 2822 | -0.510 | -0.4519 | Yes |
| 238 | ASF1B |  |  | 2824 | -0.510 | -0.4502 | Yes |
| 239 | COX6C |  |  | 2825 | -0.511 | -0.4481 | Yes |
| 240 | CDCA3 |  |  | 2829 | -0.513 | -0.4470 | Yes |
| 241 | TFDP2 |  |  | 2831 | -0.514 | -0.4452 | Yes |
| 242 | TMEM97 |  |  | 2834 | -0.514 | -0.4438 | Yes |
| 243 | SPC24 |  |  | 2836 | -0.514 | -0.4420 | Yes |
| 244 | HMGB3 |  |  | 2839 | -0.516 | -0.4405 | Yes |
| 245 | TOP2A |  |  | 2842 | -0.517 | -0.4391 | Yes |
| 246 | ZNF43 |  |  | 2844 | -0.517 | -0.4373 | Yes |
| 247 | E2F8 |  |  | 2847 | -0.519 | -0.4358 | Yes |
| 248 | LIG1 |  |  | 2848 | -0.520 | -0.4337 | Yes |
| 249 | WDHD1 |  |  | 2853 | -0.522 | -0.4329 | Yes |
| 250 | CKAP2L |  |  | 2857 | -0.523 | -0.4317 | Yes |
| 251 | NUDT1 |  |  | 2859 | -0.524 | -0.4299 | Yes |
| 252 | RFC5 |  |  | 2862 | -0.526 | -0.4284 | Yes |
| 253 | CDK2 |  |  | 2863 | -0.526 | -0.4263 | Yes |
| 254 | RAD21 |  |  | 2866 | -0.527 | -0.4248 | Yes |
| 255 | NUP62 |  |  | 2867 | -0.527 | -0.4226 | Yes |
| 256 | HJURP |  |  | 2869 | -0.527 | -0.4208 | Yes |
| 257 | POLR3K |  |  | 2872 | -0.528 | -0.4193 | Yes |
| 258 | ANP32E |  |  | 2877 | -0.530 | -0.4185 | Yes |
| 259 | PTTG1 |  |  | 2895 | -0.540 | -0.4217 | Yes |
| 260 | SPECC1 |  |  | 2909 | -0.546 | -0.4237 | Yes |
| 261 | HNRNPUL1 |  |  | 2913 | -0.549 | -0.4225 | Yes |
| 262 | FAM111A |  |  | 2917 | -0.552 | -0.4212 | Yes |
| 263 | BEST3 |  |  | 2918 | -0.553 | -0.4189 | Yes |
| 264 | ARHGAP11A |  |  | 2921 | -0.556 | -0.4173 | Yes |
| 265 | TRA2B |  |  | 2933 | -0.563 | -0.4186 | Yes |
| 266 | ATAD5 |  |  | 2935 | -0.563 | -0.4166 | Yes |
| 267 | FANCA |  |  | 2937 | -0.565 | -0.4146 | Yes |
| 268 | H2AFY |  |  | 2942 | -0.568 | -0.4136 | Yes |
| 269 | OLA1 |  |  | 2946 | -0.569 | -0.4123 | Yes |
| 270 | DSN1 |  |  | 2949 | -0.571 | -0.4106 | Yes |
| 271 | KIF4A |  |  | 2953 | -0.574 | -0.4092 | Yes |
| 272 | PBX3 |  |  | 2959 | -0.578 | -0.4085 | Yes |
| 273 | KIF23 |  |  | 2961 | -0.579 | -0.4065 | Yes |
| 274 | H1FX |  |  | 2968 | -0.581 | -0.4060 | Yes |
| 275 | NCAPH |  |  | 2974 | -0.585 | -0.4053 | Yes |
| 276 | GGH |  |  | 2975 | -0.585 | -0.4029 | Yes |
| 277 | CTNNAL1 |  |  | 2976 | -0.585 | -0.4005 | Yes |
| 278 | VRK1 |  |  | 2983 | -0.592 | -0.4000 | Yes |
| 279 | SMC2 |  |  | 2984 | -0.592 | -0.3976 | Yes |
| 280 | SKA3 |  |  | 2985 | -0.592 | -0.3952 | Yes |
| 281 | ILDR2 |  |  | 2990 | -0.595 | -0.3941 | Yes |
| 282 | TPX2 |  |  | 3006 | -0.606 | -0.3965 | Yes |
| 283 | C19orf48 |  |  | 3013 | -0.608 | -0.3959 | Yes |
| 284 | TCF19 |  |  | 3019 | -0.613 | -0.3950 | Yes |
| 285 | RTKN2 |  |  | 3020 | -0.613 | -0.3926 | Yes |
| 286 | ZEB2 |  |  | 3031 | -0.620 | -0.3933 | Yes |
| 287 | TOPBP1 |  |  | 3033 | -0.621 | -0.3911 | Yes |
| 288 | DNMT1 |  |  | 3035 | -0.622 | -0.3888 | Yes |
| 289 | LSM3 |  |  | 3037 | -0.625 | -0.3866 | Yes |
| 290 | PARP1 |  |  | 3039 | -0.626 | -0.3844 | Yes |
| 291 | RBBP8 |  |  | 3053 | -0.637 | -0.3860 | Yes |
| 292 | AURKB |  |  | 3056 | -0.639 | -0.3841 | Yes |
| 293 | LMNB2 |  |  | 3061 | -0.644 | -0.3827 | Yes |
| 294 | NCAPD3 |  |  | 3064 | -0.646 | -0.3807 | Yes |
| 295 | ALYREF |  |  | 3068 | -0.649 | -0.3791 | Yes |
| 296 | FANCI |  |  | 3074 | -0.656 | -0.3780 | Yes |
| 297 | C21orf58 |  |  | 3078 | -0.659 | -0.3763 | Yes |
| 298 | CDC45 |  |  | 3081 | -0.661 | -0.3743 | Yes |
| 299 | FBXW7 |  |  | 3082 | -0.662 | -0.3716 | Yes |
| 300 | GTSE1 |  |  | 3083 | -0.663 | -0.3689 | Yes |
| 301 | ZNF704 |  |  | 3086 | -0.665 | -0.3668 | Yes |
| 302 | TMPO |  |  | 3088 | -0.666 | -0.3644 | Yes |
| 303 | MAD2L1 |  |  | 3099 | -0.675 | -0.3649 | Yes |
| 304 | NASP |  |  | 3106 | -0.682 | -0.3641 | Yes |
| 305 | RFC3 |  |  | 3108 | -0.683 | -0.3616 | Yes |
| 306 | CNBP |  |  | 3113 | -0.686 | -0.3601 | Yes |
| 307 | PA2G4 |  |  | 3116 | -0.690 | -0.3580 | Yes |
| 308 | DBF4 |  |  | 3117 | -0.690 | -0.3551 | Yes |
| 309 | MYBL1 |  |  | 3119 | -0.691 | -0.3527 | Yes |
| 310 | LMNB1 |  |  | 3121 | -0.692 | -0.3502 | Yes |
| 311 | POLD3 |  |  | 3122 | -0.695 | -0.3473 | Yes |
| 312 | PRR11 |  |  | 3125 | -0.698 | -0.3451 | Yes |
| 313 | SMC4 |  |  | 3126 | -0.699 | -0.3423 | Yes |
| 314 | CENPK |  |  | 3127 | -0.700 | -0.3395 | Yes |
| 315 | RFC4 |  |  | 3129 | -0.701 | -0.3369 | Yes |
| 316 | MCM5 |  |  | 3132 | -0.706 | -0.3347 | Yes |
| 317 | GINS1 |  |  | 3137 | -0.713 | -0.3331 | Yes |
| 318 | GMNN |  |  | 3140 | -0.719 | -0.3308 | Yes |
| 319 | KIF15 |  |  | 3142 | -0.723 | -0.3282 | Yes |
| 320 | BTG3 |  |  | 3148 | -0.727 | -0.3268 | Yes |
| 321 | CDK1 |  |  | 3149 | -0.727 | -0.3239 | Yes |
| 322 | HNRNPD |  |  | 3157 | -0.738 | -0.3231 | Yes |
| 323 | MKI67 |  |  | 3158 | -0.738 | -0.3201 | Yes |
| 324 | ORC6 |  |  | 3159 | -0.738 | -0.3171 | Yes |
| 325 | PRIM1 |  |  | 3160 | -0.739 | -0.3141 | Yes |
| 326 | TK1 |  |  | 3162 | -0.745 | -0.3114 | Yes |
| 327 | DBI |  |  | 3165 | -0.746 | -0.3090 | Yes |
| 328 | RNASEH2B |  |  | 3166 | -0.747 | -0.3060 | Yes |
| 329 | DNAJC9 |  |  | 3167 | -0.747 | -0.3029 | Yes |
| 330 | STMN1 |  |  | 3171 | -0.751 | -0.3009 | Yes |
| 331 | DCHS1 |  |  | 3173 | -0.751 | -0.2981 | Yes |
| 332 | OXCT1 |  |  | 3176 | -0.756 | -0.2957 | Yes |
| 333 | MCM8 |  |  | 3178 | -0.758 | -0.2929 | Yes |
| 334 | MCM3 |  |  | 3182 | -0.761 | -0.2908 | Yes |
| 335 | USP1 |  |  | 3183 | -0.762 | -0.2877 | Yes |
| 336 | BRCA1 |  |  | 3185 | -0.765 | -0.2849 | Yes |
| 337 | CDCA2 |  |  | 3189 | -0.767 | -0.2828 | Yes |
| 338 | SALL1 |  |  | 3190 | -0.768 | -0.2796 | Yes |
| 339 | MAGI1 |  |  | 3193 | -0.771 | -0.2771 | Yes |
| 340 | XRCC2 |  |  | 3197 | -0.777 | -0.2750 | Yes |
| 341 | TMA7 |  |  | 3198 | -0.780 | -0.2718 | Yes |
| 342 | BARD1 |  |  | 3201 | -0.783 | -0.2692 | Yes |
| 343 | CDCA7 |  |  | 3203 | -0.785 | -0.2664 | Yes |
| 344 | CDC6 |  |  | 3204 | -0.785 | -0.2632 | Yes |
| 345 | BIRC5 |  |  | 3208 | -0.793 | -0.2609 | Yes |
| 346 | FBXO5 |  |  | 3212 | -0.797 | -0.2586 | Yes |
| 347 | DIAPH3 |  |  | 3213 | -0.800 | -0.2554 | Yes |
| 348 | RRM1 |  |  | 3215 | -0.801 | -0.2524 | Yes |
| 349 | SPC25 |  |  | 3219 | -0.806 | -0.2501 | Yes |
| 350 | DLX6 |  |  | 3223 | -0.810 | -0.2478 | Yes |
| 351 | MDK |  |  | 3224 | -0.811 | -0.2445 | Yes |
| 352 | UBE2C |  |  | 3225 | -0.812 | -0.2412 | Yes |
| 353 | SYNE2 |  |  | 3231 | -0.820 | -0.2395 | Yes |
| 354 | BCHE |  |  | 3236 | -0.825 | -0.2374 | Yes |
| 355 | NUSAP1 |  |  | 3237 | -0.825 | -0.2341 | Yes |
| 356 | FOXM1 |  |  | 3238 | -0.826 | -0.2307 | Yes |
| 357 | TMSB15A |  |  | 3241 | -0.833 | -0.2279 | Yes |
| 358 | HMGN2 |  |  | 3242 | -0.833 | -0.2246 | Yes |
| 359 | RFC2 |  |  | 3245 | -0.839 | -0.2218 | Yes |
| 360 | LITAF |  |  | 3247 | -0.843 | -0.2187 | Yes |
| 361 | DTL |  |  | 3249 | -0.848 | -0.2156 | Yes |
| 362 | PFN2 |  |  | 3252 | -0.852 | -0.2127 | Yes |
| 363 | NRXN3 |  |  | 3254 | -0.854 | -0.2096 | Yes |
| 364 | RIC3 |  |  | 3265 | -0.873 | -0.2093 | Yes |
| 365 | CENPM |  |  | 3269 | -0.881 | -0.2066 | Yes |
| 366 | CHAF1A |  |  | 3270 | -0.882 | -0.2031 | Yes |
| 367 | HUNK |  |  | 3272 | -0.884 | -0.1998 | Yes |
| 368 | CDCA5 |  |  | 3273 | -0.885 | -0.1962 | Yes |
| 369 | RHOBTB3 |  |  | 3275 | -0.887 | -0.1929 | Yes |
| 370 | CCNE2 |  |  | 3276 | -0.889 | -0.1893 | Yes |
| 371 | FEN1 |  |  | 3278 | -0.890 | -0.1860 | Yes |
| 372 | H2AFX |  |  | 3282 | -0.898 | -0.1833 | Yes |
| 373 | HELLS |  |  | 3284 | -0.901 | -0.1799 | Yes |
| 374 | RANBP1 |  |  | 3290 | -0.911 | -0.1779 | Yes |
| 375 | ZWINT |  |  | 3296 | -0.925 | -0.1757 | Yes |
| 376 | HMGA1 |  |  | 3299 | -0.931 | -0.1726 | Yes |
| 377 | FBLN1 |  |  | 3317 | -0.954 | -0.1742 | Yes |
| 378 | PCNA |  |  | 3319 | -0.963 | -0.1706 | Yes |
| 379 | UBE2T |  |  | 3330 | -0.979 | -0.1698 | Yes |
| 380 | MYBL2 |  |  | 3340 | -1.006 | -0.1686 | Yes |
| 381 | MCM4 |  |  | 3341 | -1.006 | -0.1645 | Yes |
| 382 | EZH2 |  |  | 3342 | -1.015 | -0.1604 | Yes |
| 383 | DEK |  |  | 3344 | -1.025 | -0.1566 | Yes |
| 384 | RAD51AP1 |  |  | 3345 | -1.030 | -0.1524 | Yes |
| 385 | PROM1 |  |  | 3347 | -1.035 | -0.1485 | Yes |
| 386 | PBK |  |  | 3354 | -1.046 | -0.1462 | Yes |
| 387 | MCM10 |  |  | 3356 | -1.050 | -0.1422 | Yes |
| 388 | CDK6 |  |  | 3366 | -1.085 | -0.1407 | Yes |
| 389 | ESCO2 |  |  | 3367 | -1.088 | -0.1363 | Yes |
| 390 | NCAPG |  |  | 3370 | -1.098 | -0.1325 | Yes |
| 391 | MCM7 |  |  | 3384 | -1.147 | -0.1320 | Yes |
| 392 | GINS2 |  |  | 3386 | -1.156 | -0.1276 | Yes |
| 393 | DHFR |  |  | 3387 | -1.159 | -0.1229 | Yes |
| 394 | CHD7 |  |  | 3389 | -1.167 | -0.1185 | Yes |
| 395 | HMGB2 |  |  | 3390 | -1.184 | -0.1137 | Yes |
| 396 | CLSPN |  |  | 3391 | -1.184 | -0.1088 | Yes |
| 397 | SOX2 |  |  | 3394 | -1.198 | -0.1046 | Yes |
| 398 | RAB3IP |  |  | 3395 | -1.198 | -0.0997 | Yes |
| 399 | CENPU |  |  | 3398 | -1.230 | -0.0954 | Yes |
| 400 | HIST1H1E |  |  | 3401 | -1.237 | -0.0910 | Yes |
| 401 | FAM111B |  |  | 3402 | -1.238 | -0.0859 | Yes |
| 402 | ATAD2 |  |  | 3415 | -1.278 | -0.0846 | Yes |
| 403 | RRM2 |  |  | 3420 | -1.297 | -0.0806 | Yes |
| 404 | HIST1H1C |  |  | 3427 | -1.335 | -0.0771 | Yes |
| 405 | HES6 |  |  | 3431 | -1.366 | -0.0726 | Yes |
| 406 | TYMS |  |  | 3436 | -1.391 | -0.0682 | Yes |
| 407 | AKAP12 |  |  | 3444 | -1.501 | -0.0643 | Yes |
| 408 | DLX1 |  |  | 3455 | -1.622 | -0.0610 | Yes |
| 409 | PRDX2 |  |  | 3462 | -1.713 | -0.0559 | Yes |
| 410 | HIST1H1D |  |  | 3466 | -1.743 | -0.0498 | Yes |
| 411 | HIST1H1B |  |  | 3467 | -1.770 | -0.0426 | Yes |
| 412 | DLX2 |  |  | 3474 | -1.814 | -0.0372 | Yes |
| 413 | CDCA7L |  |  | 3481 | -1.878 | -0.0315 | Yes |
| 414 | ASCL1 |  |  | 3484 | -1.937 | -0.0242 | Yes |
| 415 | HIST1H4C |  |  | 3493 | -2.248 | -0.0177 | Yes |
| 416 | CKB |  |  | 3497 | -2.376 | -0.0090 | Yes |
| 417 | LMO1 |  |  | 3507 | -3.235 | 0.0013 | Yes |
Table: GSEA details [plain text format]

  

Fig 2: RICHARDS\_NOWAKOWSKI\_SCIENCE\_2017\_MGE\_DIV\_UPREG: Random ES distribution      
 Gene set null distribution of ES for **RICHARDS\_NOWAKOWSKI\_SCIENCE\_2017\_MGE\_DIV\_UPREG**
